# Letter from a Sick Room

**Published:** 1854-04

**Authors:** 


### LETTER FROM A SICK ROOM.

"When I tell you, gentlemen, that I am a poor sick girl, propped up in my easy chair, scribbling to you, no doubt you will marvel that I make this exertion, when I can probably say so little to interest either you or your readers. True enough! But as I declare my object to be a simple desire to thank you for that *Budget of Goodies*, received weekly, I trust you will pardon my boldness. No matter what the weather may be! Does the wind blow? Or the storm come pelting upon my casement? Does my ninth bottle of "Cod Liver Oil" stand staring me in the face? Does "Croton" and "Mustard" nip and bite? Or do sad thoughts steal into my heart, and my little chamber seem dark and solitary? All these evils are forgotten when I hear a light tap upon the door, and "Josey" enters bearing the *Home Journal*, and the "Dr.'s compliments, Miss," and "hopes you are quite comfortable." Down goes the "oil!" and away scamper "dark clouds" and "sad thoughts!" while my soul enjoys refreshing draughts from your columns. I like everything you say, but especially the hints you drop now and then about those young city ladies who frequent gay scenes, dressing in the most extravagant and ridiculous manner imaginable! Poor silly creatures! But who can blame them, while their simple old "mammas" encourage them in such foolishness! *They* surely ought to know better; for were they *honestly* to consult their own situation, physically and mentally, they could but acknowledge that an aching heart and body must be the result, if not premature death. But where, in the name of health and common sense, are the fathers and brothers of these little simpletons? If their mothers are not to be depended upon, why do not those who secretly confess the revolting impression a half-dressed, dissipated, sickly female produces upon their minds,—I say, why do *they* not forbid such wickedness? Why do they not refuse to accompany, or permit them to go to scenes of amusements or elsewhere, unless comfortably and decently attired? Ah! Echo answers—"*Why*" *!!!* You will, perhaps, deem my remarks somewhat severe, but as I sit here in my "easy-chair," my thoughts wander back to past follies and imprudencies, which I would give worlds to recall! "Thin shoes,"

and "damp feet," repeated "coughs" and "colds," flit before me! Warnings came, and passed unheeded, and in *bitterness* am I reaping the reward of such negligence! Excluded from the world, and often even from personal interviews with dear friends, as excitement and over-talking soon place me upon my sofa, and I lose all I seemed to have gained. "Consumption!" (that fell destroyer,) seems hovering near, and I may well fear him, for already has he robbed us of many dear ones. Once we numbered seven, but now, alas! *three* are resting in yonder little enclosure. While the dark-eyed "Lizzie" reposes in her native New England, the "first-born" loving and true-hearted "Mary," sleeps far away in the "sunny South." But Death did not stop here! One other! noble and near of kin! bound to our affections by cords that never can be broken, left our home one bright summer day, full of hope, and promising speedy return. But the faithful "Missionary" in a far-distant island of the Pacific, soothed his dying couch, and received his parting sigh! Never shall I forget that morning the sad news reached us that another loved one had gone down to the grave! By the last steamer, had been received letters of happy anticipations, bidding us look forward a few short months, when he would return, and with him a fair bride, whose happiness it should be to supply, as far as possible, the blank left in our hearts by past sorrow. With joy we penned answers full of love and welcome! A few hours later, a black-sealed missive was handed us, and we learned that a "miniature," a "lock of hair," and a few "withered flowers" plucked from his grave, were all we had to look upon of my once-loved brother. A year has passed since then, and now *my* turn has come to suffer, and perhaps *die*, ere the rose shall blossom again. One object binds me strongly to earth! A dear old *father* still lingers with me, trembling upon the verge of eternity, as though unwilling to leave me *quite alone!* He is *so* gentle, *so* kind; and his eye follows me as tenderly as that of a fond mother, and seems to say, "I live but for you," while my poor heart echoes back, "*I* but for *you*, dear father!" And now, dear sirs, I must close this "epistle from my easy-chair." Once more let me thank you for your delightful paper, and through you, my good physician, by whose politeness I receive it, and who displays not a little shrewdness in his prescription of the *Home Journal*, in place of useless concoctions and disgustingly crammed *pill boxes !* Yours, truly,

LOUISE.

The above letter is taken from the "*Home Journal*," printed weekly in New York, for two dollars a year, a paper which few subscribers cease to take, as long as it is possible to spare the subscription price, beautiful in its mechanical execution, regular in its appearance, sparkling, varied, and always interesting.

Was that "poor sick girl's" condition a necessity, or the result of a neglected physical education ?

Louise ! is your mother's bible near ? *it* will give enduring comfort.

